# Characterization of a liposomal copper(II)-quercetin formulation suitable for parenteral use

**DOI:** 10.1007/s13346-019-00674-7

**Published:** 2019-09-03

**Authors:** Kent T. J. Chen, Malathi Anantha, Ada W. Y. Leung, Jayesh A. Kulkarni, Gardenia G. C. Militao, Mohamed Wehbe, Brent Sutherland, Pieter R. Cullis, Marcel B. Bally

**Affiliations:** 1 Department of Experimental Therapeutics, BC Cancer Research, Vancouver, BC V5Z 1L3 Canada; 2 Department of Interdisciplinary Oncology, BC Cancer Research, Vancouver, BC V5Z 1L3 Canada; 3 Department of Pathology and Laboratory Medicine, University of British Columbia, Vancouver, BC V6T 2B5 Canada; 4 Cuprous Pharmaceuticals, Vancouver, BC V6N 3P8 Canada; 5 Department of Medical Genetics, BC Children’s Hospital Research Institute, University of British Columbia, Vancouver, BC V6T 1Z3 Canada; 6 Department of Biochemistry and Molecular Biology, University of British Columbia, Vancouver, BC V6T 1Z3 Canada; 7 Federal University of Pernambuco, Recife, PE CEP:50.670-901 Brazil; 8 Faculty of Pharmaceutical Sciences, University of British Columbia, Vancouver, BC V6T 1Z3 Canada

**Keywords:** Quercetin, Cancer, Flavonoids, Copper complexes, Liposomes

## Abstract

Quercetin (3,3′,4′,5,7-pentahydroxyflavone) is a naturally derived flavonoid that is commonly found in fruits and vegetables. There is mounting evidence to suggest that quercetin has potential anticancer effects and appears to interact synergistically when used in combination with approved chemotherapeutic agents such as irinotecan and cisplatin. Unfortunately, quercetin has shown limited clinical utility, partly due to low bioavailability related to its poor aqueous solutions (< 10 μg/mL). In this study, liposomal formulations of quercetin were developed by exploiting quercetin’s ability to bind copper. Quercetin powder was added directly to pre-formed copper-containing liposomes (2-distearoyl-sn-glycero-3-phosphocholine (DSPC) and cholesterol (CHOL) (55:45 M ratio)). As a function of time and temperature, the formation of copper-quercetin was measured. Using this methodology, a final quercetin-to-lipid (mol:mol) ratio of 0.2 was achievable and solutions containing quercetin at concentrations of > 5 mg/mL were attained, representing at least a > 100-fold increase in apparent solubility. The resulting formulation was suitable for intravenous dosing with no overt toxicities when administered at doses of 50 mg/kg in mice. Pharmacokinetic studies demonstrated that the copper-quercetin formulations had an AUC_0-24H_ of 8382.1 μg h/mL when administered to mice at 50 mg/kg. These studies suggested that quercetin (not copper-quercetin) dissociates from the liposomes after administration. The resulting formulation is suitable for further development and also serves as a proof-of-concept for formulating other flavonoids and flavonoid-like compounds. Given that quercetin exhibits an IC_50_ of >10 μM when tested against cancer cell lines, we believe that the utility of this novel quercetin formulation for cancer indications will ultimately be as a component of a combination product.

## Introduction

One of the most common phenolics found in fruits and vegetables are the flavonoids. These phenolic compounds are believed to be one reason why fruit- and vegetable-rich diets are commonly associated with lowered risk for cancer [1]. The most common, naturally occurring flavonoid is quercetin (3,3′,4′,5,7-pentahydroxyflavone) and it has garnered much attention as a potential anticancer agent [2, 3]. Quercetin is well-established as an effective antioxidant, protecting against damage associated with oxidative stress from free radicals or reactive oxidative species (ROS) [4, 5]. Specifically, quercetin’s antioxidant activity may be due to catalase’s ability to protect cells from oxidative stress and damage by scavenging H_2_O_2_ [4–6]. It has also been suggested that quercetin exhibits anticancer activity through mechanisms that involve induction of apoptosis. In particular, studies with A549 lung cancer cells, human glioma cells, and human hepatoma cells (HepG2) have shown that treatment with quercetin can concurrently downregulate anti-apoptotic proteins such as Bcl-2, AKT, and metallopeptidase 9 and upregulate pro-apoptotic proteins such as Bax [3, 7, 8].

Quercetin’s potential as an anticancer agent is further demonstrated in combination therapy. When used in a combination setting, quercetin appears to enhance cisplatin cytotoxicity against ovarian cancer in various murine models of cancer [2]. The use of quercetin may also reduce cisplatin-induced ototoxicity as it was shown to protect zebrafish embryos against sensory hair cell damage engendered by cisplatin [9].

Due to its poor aqueous solubility, most applications of quercetin, whether in the context of clinical trials or as a supplement to therapy, rely on oral administration. However, poor dissolution in gastrointestinal fluids leads to poor gastrointestinal absorption of quercetin, resulting in low bioavailability [10]. Therefore, despite its potential chemotherapeutic benefit, the clinical use of quercetin as an anticancer agent has remained essentially irrelevant [11, 12]. To fully explore the therapeutic potential of quercetin, there is a need to improve its solubility and various strategies have been used to achieve this including the development of more water-soluble pro-quercetin compounds that biologically convert to quercetin [13] as well as various formulation approaches involving the use of liposomes [14], polymers [15], or milling to produce nanocrystals [16]. In this study, we took advantage of quercetin’s propensity to bind copper [17–19] to develop for the first time a liposomal formulation of copper-quercetin suitable for intravenous use. The resulting formulation exhibits at least a 100-fold improvement in the apparent solubility of quercetin and is suitable for intravenous administration.

## Materials and methods

### Materials

Quercetin, Sephadex G-50, Copper (II) sulfate, HEPES, ethylenediaminetetraacetic acid (EDTA), and sodium chloride (NaCl) were purchased from Sigma-Aldrich (St. Louis, MO). 1,2-Disteoroyl-sn-glycero-3-phosphatidylcholine (DSPC) and cholesterol were purchased from Avanti Polar Lipids, Inc. (Alabaster, AL, USA). ^3^[H]-cholesteryl hexadecyl ether (CHE) and picofluor-15 scintillation fluid were obtained from PerkinElmer, Inc. (Boston, MA, USA). Copper (II) gluconate powder was purchased from AlfaAesar (Ward Hill, MA, USA). Sucrose was obtained from EMD Chemicals Inc. (Gibbstown, NJ, USA). Citric acid monohydrate crystals were purchased from EmScience (USA).

### Determination of quercetin solubility

Two milliliters of Milli-Q water, 300 mM sucrose/20 mM HEPES (pH 7.4) (SH), or 150 mM NaCl/20 mM HEPES (pH 7.4) (HBS) was added to 10 mg of quercetin and the solution was subsequently mixed for 60 min at either 60 °C or room temperature (RT). Samples at 60 °C were cooled to room temperature prior to filtration through 0.45-μm filters to remove undissolved quercetin. The resulting clear solutions were then assayed for quercetin via UV-Vis spectroscopy (absorbance at 372 nm) on the Hewlett Packard/Agilent 8453 G1103A UV-Visible Spectrophotometer (Agilent Technologies, Santa Clara, CA, USA).

### Liposome preparation

DSPC and cholesterol were dissolved in chloroform at a 55:45 mol:mol ratio. ^3^[H]-CHE was added as a non-exchangeable and non-metabolizable lipid marker [20] prior to drying. ^3^H-CHE was incorporated into the chloroform mixture at a concentration of 5.0–12.5 nanocurie per μmol lipid. Chloroform was removed by drying the samples under a steady stream of nitrogen gas until the samples became thick and syrup-like. The samples were then placed under high vacuum to create a “puff” of dried lipid which was kept under vacuum for at least 3 h to remove residual chloroform. Unless stated differently, the dried lipid was hydrated in 300 mM copper sulfate (CuSO_4_) or 100 mM copper gluconate (CuG) (both at pH 3.5) (1 mL per 50 μmol of total lipid). To study the role of pH, lipids were hydrated in 100 mM copper gluconate (pH adjusted to 7.4 with triethanolamine (TEA)). Lipid hydration results in the formation of multilamellar liposomes and the resulting multilamellar liposomes were then extruded 10 times through two stacked 0.1 μm polycarbonate filters in a 10-mL thermobarrel extruder (Extruder™, Evonik Transferra Nanosciences, Burnaby, BC, Canada). The extruded unilamellar liposomes had a mean diameter of 110 ± 20 nm as determined by Phase Analysis Light Scattering methods (ZetaPALS, Brookhaven Instruments Corp., Holtsville, NY). To remove external copper, the sample was passed through a Sephadex G50 column pre-equilibrated with SHE buffer (300 mM sucrose, 20 mM HEPES, 15 mM EDTA) adjusted to pH 7.4. The liposome-containing fractions were subsequently passed through a second Sephadex G50 column pre-equilibrated with HBS. The final lipid concentration was determined by measuring ^3^[H]-CHE using liquid scintillation counting (Packard 1900 TR Liquid Scintillation analyzer). To gain an understanding of quercetin-copper stoichiometry, liposomes were also prepared with different concentrations of either CuSO_4_ or CuG which were adjusted to control for osmolarity using sodium sulfate or sodium chloride, respectively. Copper-free liposomes were prepared by hydrating lipids in 300 mM citric acid (pH 3.5) or SH buffer (pH 7.4). In all cases, the external buffer was exchanged to HBS (pH 7.4) prior to initiating studies with added quercetin.

### Copper-quercetin formation inside liposomes

Unless otherwise specified, quercetin was added to liposomes at an initial quercetin-to-lipid ratio of 0.4 (mol/mol). The mixture was then continuously mixed with a mini stir-bar at specified temperatures ranging from RT to 60 °C and the amount of liposome-associated quercetin was measured. For these studies, 80 μL aliquots from the quercetin-liposome mixture were taken at the indicated time points and passed through 1-mL Sephadex G-50 mini-spin columns (680×*g*; 3 min) to separate free quercetin from liposome-associated quercetin. The excluded fraction, which appears yellow due to the formation of the copper-quercetin complex, was then analyzed for lipid, quercetin, and copper concentrations. Liposomal lipid (^3^[H]-CHE - tagged) was analyzed by liquid scintillation counting (10 μL of sample to 5 mL of scintillation fluid). Quercetin was measured using absorbance at 372 nm on the Hewlett Packard/Agilent 8453 G1103A UV-Visible Spectrophotometer (Agilent Technologies, Santa Clara, CA, USA) after the samples (100 μL) were diluted in acidified methanol (methanol with 3% glacial acetic acid). Copper was measured using atomic absorption spectroscopy where samples were diluted with 0.1% HNO_3_ (v/v with Milli-Q water) and analyzed using the AAnalyst 600 Atomic Absorbance Spectrometer (PerkinElmer, Inc. Woodbridge, ON, Canada): the samples were dried at 110 °C for 30s, heated (45 s) to 130 °C, ashed at 1200 °C for 30s, and atomized and cleaned at 2000 °C (5 s) and 2450 °C (4 s), respectively.

### Characterization of copper-quercetin binding

Five milligrams of quercetin powder was dissolved in 10 mL of methanol and the resulting solution was diluted with methanol to a final quercetin concentration of 5 μg/mL. Varying concentrations of CuSO_4_ and CuG were then added (10 μL) to achieve copper-to-quercetin ratios of 1:8, 1:4, 1:2, 1:1, 2:1, 4:1, and 8:1. These samples were then analyzed spectrophotometrically (Hewlett Packard/Agilent 8453 G1103A UV-Visible Spectrophotometer).

### In vitro serum stability assay

The indicated liposomal copper-quercetin formulations were prepared at 60 °C as described above and concentrated by tangential flow filtration (TFF) using an ultrafiltration hollow fiber column (molecular weight cutoff 500 kDa) (GE Healthcare) to achieve a final quercetin concentration of 16.5 mM (lipid concentration was 100 mM). Four hundred microliters of this sample was added to 1.6 mL of fetal bovine serum (FBS; Gibco, Burlington, ON, Canada) and mixed at 37 °C. At various time points (0, 1, 4, 8, and 24 h), 80 μL aliquots from the mixture were passed through mini-spin columns as described above. The excluded fraction was then diluted in acidified methanol and centrifuged at 10,000 rpm at 4 °C for 10 min to remove precipitated proteins. The resulting supernatant was collected and used to determine quercetin, copper, and liposomal lipid concentrations as described above.

### Cryogenic electron microscopy of copper-quercetin formulations

Cryogenic electron microscopy (CEM) was performed as previously described [21]. Particles were concentrated to a final concentration of ~ 20 mg/mL lipid and added to TEM grids, and vitreous ice was generated using an FEI Mark IV Vitrobot (FEI, Hillsboro, OR). Prepared grids were stored in liquid nitrogen until imaged. A Gatan 70° cryo-tilt transfer system pre-equilibrated to at least − 180 °C prior to insertion into the microscope was used as the sample holder. An FEI LaB6 G2 TEM (FEI, Hillsboro, OR) operating at 200 kV under low-dose conditions was used to image all samples. A bottom-mount FEI Eagle 4 K CCD camera was used to capture all images. All samples (unless otherwise stated) were imaged at a × 55,000 magnification with a nominal under-focus of 1–2 μm to enhance contrast. Sample preparation and imaging was performed at the UBC Bioimaging Facility (Vancouver, BC).

### Pharmacokinetics of the copper-quercetin formulations

Preliminary pharmacokinetic studies were done in Rag2-M mice obtained from the Animal Resource Centre (ARC) at BC Cancer’s Vancouver Research Centre (Vancouver, BC, Canada). All studies were conducted in accordance with the guidelines set by the Canadian Council on Animal Care, as overseen by the University of British Columbia’s Animal Care Committee (protocols A10-0171, A10-0206, and A14-0290). All mice were placed in Optimice cages (up to 5 mice per cage) and cages were equipped with nesting materials and plastic hiding structures (“huts”) for environmental enrichment. Animals were provided with food and water ad libitum. The health status of all animals used in these studies was monitored at least once daily following an established standard operating procedure designed to assess animal health status. In particular, signs of ill health were based on body weight loss, change in appetite, and behavioral changes such as altered gait, lethargy and gross manifestations of stress. When signs of severe toxicity were present, the animals were terminated (isoflurane overdose followed by CO_2_ asphyxiation) for humane reasons. Necropsy was performed to assess signs of toxicity.

For injected samples, the liposomes were prepared as described above and filter-sterilized through the sterile 50-mL vacuum-driven Steriflip filtration system (Millipore, Billerica, MA, USA). Quercetin was then added to the liposomes incubated for 30 min at 60 °C then cooled to room temperature, filtered, and exchanged into HBS buffer as described above. The resulting liposomal quercetin formulation was concentrated to 5 mg/mL via TFF as described above. The filter-sterilized formulation was then injected intravenously at a quercetin dose of 50 mg/kg (injection volume was 10 μL/g body weight).

Following iv administration, animals (4 per time point) were sacrificed (isoflurane overdose followed by CO_2_ asphyxiation) at the indicated time points and blood was collected via cardiac puncture. The blood was then centrifuged (2500 rpm; 15 min) and plasma was collected and placed in a tube. The plasma was kept at 4 °C until all analyses were completed. Lipid and copper concentrations in plasma were determined as described above. Quercetin concentrations were measured via high-performance liquid chromatography (HPLC; Waters, Milford, MA) at an absorbance wavelength of 368 nm. Specifically, plasma samples were mixed with chilled 3% acidified methanol, then centrifuged at 14,000 rpm for 10 min to pellet precipitated plasma proteins. Twenty-five microliters of the resulting supernatant was injected onto a Symmetry C_18_ column (particle size 3.5 μm; 4.6 × 75 mm, Waters). The mobile phase consisted of 70% mobile phase “A” (1% triethyleneamine in water, pH 6.4 adjusted with glacial acetic acid) and 30% mobile phase “B” (100% acetonitrile). The sample temperature was maintained at 4 °C while the column temperature was set at 30 °C. Run time was 5 min per sample with a flow rate of 1 mL/min using a gradient method with the amount of organic phase increasing from 12 to 40% over 8 min.

### In vitro cytotoxicity assays of quercetin alone and in combination with irinotecan

A549 lung adenocarcinoma cells were kindly provided by Dr. John Minna’s Laboratory (Dallas, TX) while BxPC3 pancreatic adenocarcinoma cells were purchased from ATCC (Manassas, VA). Both cell lines were cultured in RPMI-1640 medium (Life Technologies, Carlsbad, CA) supplemented with 10% fetal bovine serum (Life Technologies) and 2 mM l-glutamine (Life Technologies). All in vitro experiments were performed between the third and eighteenth passages. All cell lines were tested negative for mycoplasma.

Dose-dependent viability assays were completed using quercetin and irinotecan (CPT-11) added alone and in combinations to A549 and BxPC3 cells. Briefly, cells were seeded in 384-well plates. Twenty-four hours post seeding, cells were treated with quercetin (Q) or CPT-11(C) alone and in combination at a ratio of 1:2.5 (C:Q) on A549 cells and 1:18 (C:Q) on BxPC3 cells. These ratios were selected based on the ratio of the IC_50_ of each drug when used alone. Cell viability was determined 72 h post-treatment using the IN Cell Analyzer 2200 (GE Healthcare, Washington, USA) as described previously [22]. In brief, cells were stained with Hoechst for total nuclei count and ethidium homodimer I (Biotium) for dead cells at 72 h following treatment and imaged with the INCell Analyzer after a 20-min incubation at 37 °C. Cells were classified as “dead” if they showed > 30% overlap of the two stains.

Dose-response curves were constructed using Prism 6.0 (GraphPad Software, La Jolla, CA, USA) by plotting mean ± SEM from at least three independent experiments. IC_50_ values were interpolated from fitted curves. Combination indices (CI) were derived from the dose-response curves using CompuSyn software (ComboSyn, Inc., Paramus, NJ, USA). CI values > 2 were considered as antagonistic; CI values < 0.8 were considered synergistic. All other CI values were considered additive.

### Statistical analysis

All data are plotted using the Prism 6.0 software (Graphpad) as mean ± standard error of the mean (SEM). Pharmacokinetics data were analyzed for the elimination half-life and total drug exposure (area under the curve; AUC) using the PK Solutions software (Summit Research Services, USA).

## Results

### Formation of copper-quercetin within copper-containing DSPC/Chol (55:45 mol%) liposomes

**Fig. 1:**
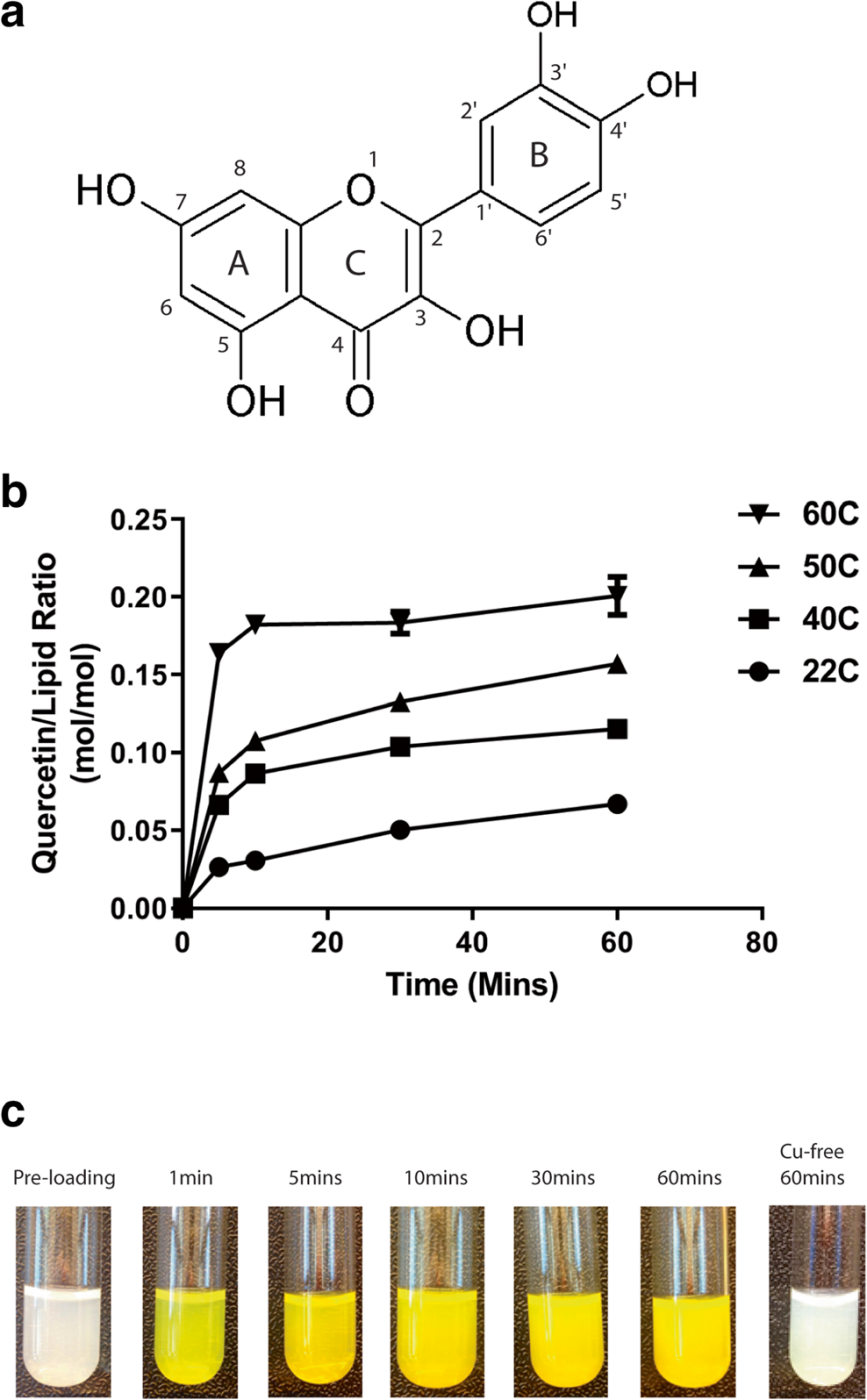
Temperature-dependent formation of copper-quercetin complexes in liposomes. **a** Quercetin (3,3′,4′,5,7-pentahydroxyflavone) is a three-ringed flavonoid. **b** The association of quercetin with 300 mM CuSO_4_-containing liposomes was assessed as a function of time (5, 10, 30, and 60 min) and temperature (22 °C, 40 °C, 50 °C, and 60 °C) after addition of the liposomes to quercetin as a powder. The results, plotted as the quercetin-to-lipid ratio (mol/mol), were determined after the mixture was eluded through a Sephadex G-50 column (see “Materials and Methods”). Data points represent the mean ± SEM (*n* = 3, where *n* = number of technical replicates). **c** Photograph of liposome solutions collected following addition of quercetin to 300 mM CuSO_4_-containing liposomes, which were then subsequently mixed at 60 °C for 60 min and eluded through a Sephadex G-50 column. The color change is consistent with formation of copper-quercetin complexes. The samples were cooled to room temperature prior to the pictures being taken

Quercetin is a triple-ringed flavonoid with capacity to coordinate copper at three domains: 3′,4′-dihydroxy group on the B ring, 3-hydroxy and 4-carbonyl group in the C ring, and the 5-hydroxy and 4-carbonyl group that spans across the A and C rings (Fig. 1a). We confirmed that quercetin exhibits limited aqueous solubility despite heating the mixtures to 60 °C (solubility is 12.33 μg/mL in water (60 °C) and 38 μg/mL in HBS (60 °C); data not shown). As described previously, the ability to form copper-quercetin within copper-containing liposomes is dependent on quercetin permeation across the liposomal lipid bilayer and this process is influenced by temperature [23]. Further, the rate of copper-quercetin complex formation is also likely temperature-dependent. Thus, the ability to prepare copper-quercetin complexes within liposomes containing copper sulfate was measured at 22 °C, 40 °C, 50 °C, and 60 °C. The results (Fig. 1b) indicate that quercetin association with the liposomes was greatest when the samples were incubated at 60 °C, where a 0.18 quercetin-to-lipid ratio (mol/mol) was achieved within 10 min and the maximum achievable ratio (0.20) was reached within 60 min. At 60 min, measured quercetin-to-lipid ratios (mol/mol) of 0.16, 0.12, and 0.07 were achieved when the samples were incubated at 50 °C, 40 °C, and 22 °C, respectively. During the incubation period, there was a color change from white to yellow (Fig. 1c), consistent with the formation of copper-quercetin following addition of the copper-containing liposomes to quercetin powder. As shown in Table 1, incubating the liposomes in the absence of quercetin at temperatures as high as 60 °C for as long as 60 min had no effects on the stability of the liposomes as indicated by the absence of particle size change.

**Table 1:** Particle size (nm) of CuSO_4_ liposomes and CuG liposomes incubated at 60 °C for 60 min in the absence or presence of quercetin

	Before incubation	After incubation
Lipo-CuSO_4_-Q	100 ± 10	140 ± 20
Lipo-CuG-Q	100 ± 10	160 ± 20
Lipo-CuSO_4_	100 ± 10	100 ± 10
Lipo-CuG	100 ± 10	100 ± 10

**Fig. 2:**
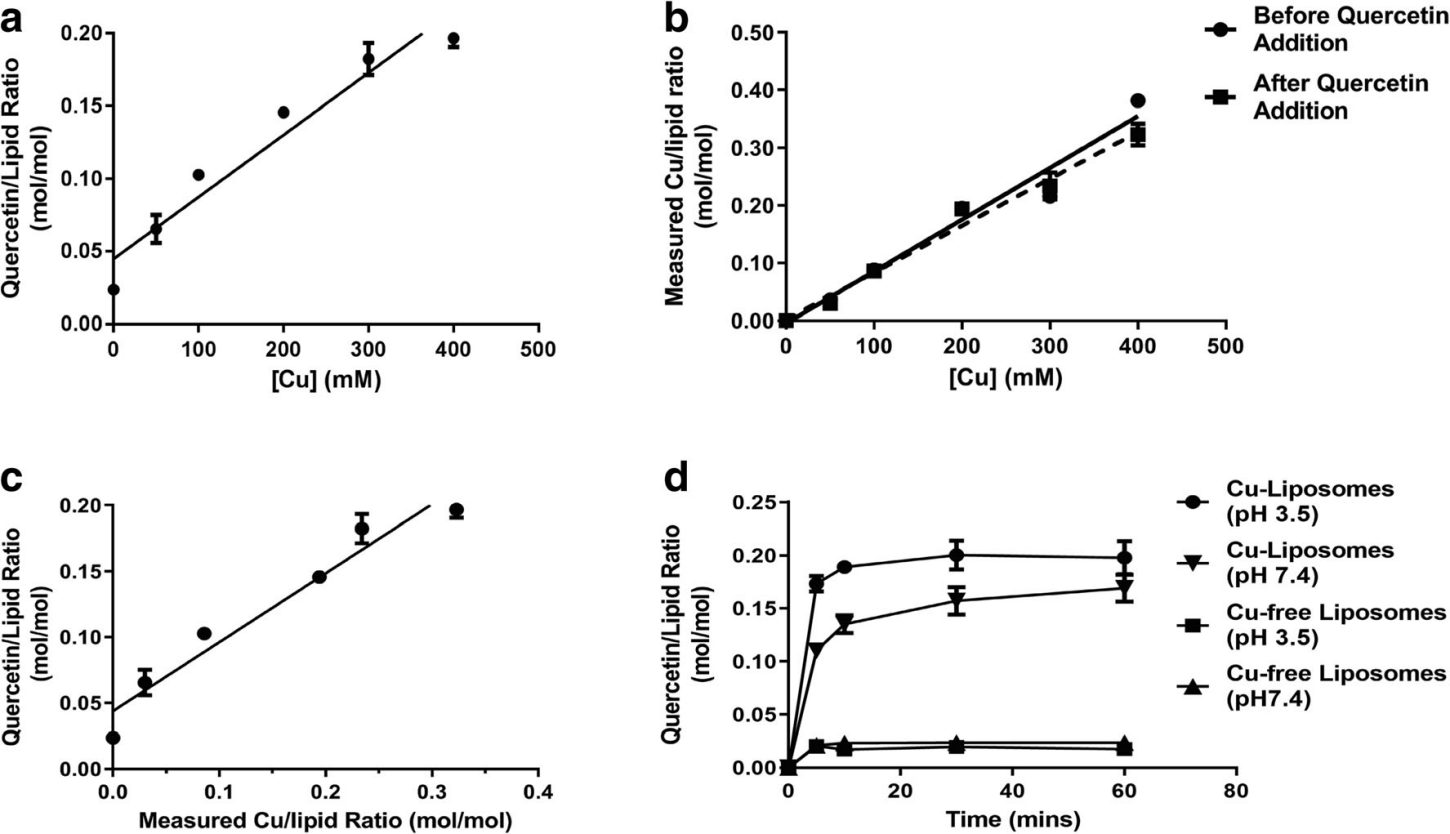
Copper-dependent quercetin association with liposomes and the impact of intra-liposomal pH. **a** Liposomes prepared in solutions containing various CuSO_4_ concentrations (0, 50, 100, 200, 300, and 400 mM CuSO_4_) were mixed with powdered quercetin (see “Materials and Methods”) at 60 °C and the samples were incubated for 60 min. The results were plotted as quercetin-to-lipid ratios. **b** Measured copper-to-lipid ratio was plotted against various CuSO_4_ concentrations. Copper was measured by AAS following separation of the quercetin liposomes on a Sephadex G-50 column. **c** The level of liposome-associated quercetin was plotted against the measured copper-to-lipid ratio. **d** Quercetin association with liposomes prepared in 100 mM copper gluconate adjusted to pH 3.5 (filled circles) or 7.4 (inverted triangles). Copper-free liposome controls were prepared in solutions containing 300 mM citric acid (adjusted to pH 3.5) or HBS (7.4) All liposomes were exchanged into HBS (pH 7.4) as described in the “Materials and Methods” section. Data points represent mean ± SEM (*n* = 3, where *n* is the number of technical replicates)

Other liposomal formulations of quercetin have been described where quercetin is added during liposome formation and quercetin is solubilized by partitioning into the liposomal membrane [24, 25]. Therefore, to demonstrate that quercetin association with the copper-containing liposomes was actually copper-dependent, several experiments were completed and these are summarized in Fig. 2. When quercetin powder was mixed with liposomes prepared using 50, 100, 200, 300, or 400 mM copper sulfate and incubated at 60 °C for 60 min, there was a copper concentration-dependent association of quercetin with the liposomes (Fig. 2a–c). The quercetin-to-lipid ratio increased with increasing CuSO_4_ concentrations and the maximum quercetin-to-lipid ratio of 0.2 was achieved using liposomes prepared in 300 mM and 400 mM copper sulfate. The results in Fig. 2b showed the calculated copper-to-lipid ratios measured for the various formulations used before and after the addition of quercetin (Fig. 2b) and these results demonstrate that no copper was released from the liposomes following quercetin addition. As suggested by the results in Fig. 2c, there was a linear relationship (*r* = 0.95) between the calculated copper-to-lipid ratio and the calculated quercetin-to-lipid ratio, with a slope of 0.52 and a *y*-axis intercept of 0.044 quercetin-to-lipid ratio. The slope suggests that a 1:2 quercetin-to-copper complex was formed within the liposomes and it can be suggested that the y-intercept represents the amount of quercetin partitioning into the liposomal lipid bilayer under the conditions described.

Additional studies were completed to assess the use of liposomes prepared in copper-free solutions and the role of using the low pH (pH 3.5) of the internal copper sulfate solution. The results, summarized in Fig. 2d, indicate low levels of quercetin association with copper-free liposome prepared using 300 mM citric acid (pH 3.5) or HBS (pH 7.4); the quercetin-to-lipid ratio was less than 0.025 which was lower than predicted based on the extrapolation made using the data in Fig. 2a. However, these data indicate that the amount of quercetin partitioning into the liposomal membrane accounts for less than 15% of the level obtained when using liposomes prepared in copper sulfate. The results also demonstrate that the level of partitioning into the lipid bilayer was not influenced by the low pH within the liposomes.

When using liposomes prepared in a pH 7.4 copper-containing solution (100 mM copper gluconate adjusted to pH 7.4 with TEA, see methods), the rate of quercetin association was lower and the maximum quercetin-to-lipid ratio achieved was lower when compared to the quercetin-to-lipid ratio achieved when using a pH 3.5 copper-containing solutions, even when adjusting for the lower copper concentrations (100 mM). However, the results, summarized in Fig. 2d, suggest that the rate of quercetin association with the CuG liposomes was influenced by the internal pH; the rate of association was faster at pH 3.5. Furthermore, Fig. 2d reiterates that quercetin association with the liposomes is dependent on the presence of encapsulated copper, but shows that the copper salt used inside the liposome affects quercetin association levels. The measured quercetin-to-lipid ratios (mol/mol) for the liposomes prepared using 100 mM CuG were as high as 0.2 and this was 2 times higher than that predicted based on liposomes prepared in CuSO_4_ solutions.

**Fig. 3:**
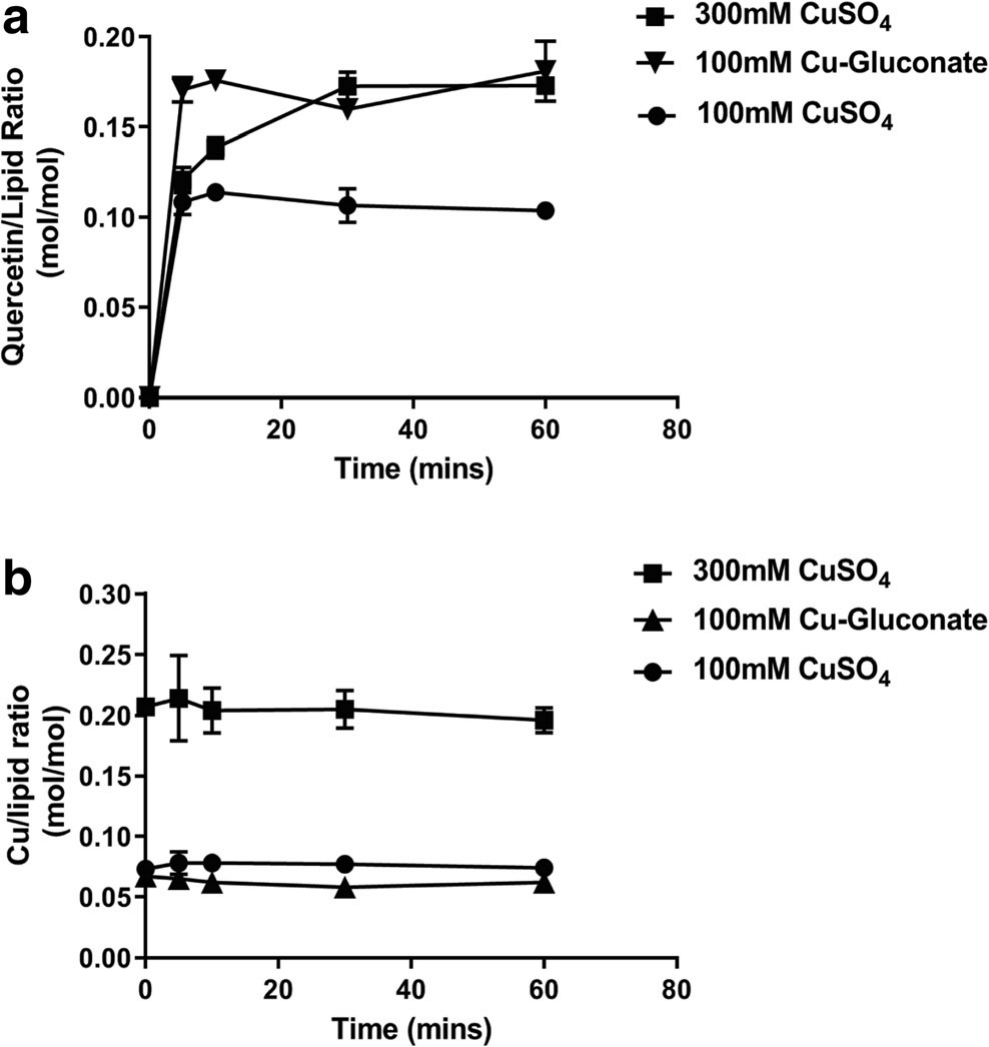
Quercetin association with liposomes prepared with CuSO_4_ or Cu-gluconate. **a** Liposomes prepared in 100 mM CuSO_4_ (filled circles), 300 mM CuSO_4_ (filled squares), and 100 mM copper gluconate (filled triangles) (all pH 3.5) were mixed with powdered quercetin at 60 °C and at the indicated time points, liposomes were passed through Sephadex G-50 spin columns (see “Materials and Methods”) and the concentration of quercetin and liposomal lipid was measured (see “Materials and Methods”) and plotted as the quercetin to lipid ratio. **b** At the same time points shown in panel **a**, the eluted liposomes were also assayed for copper by AAS (see “Materials and Methods”) and the results plotted as the copper-to-lipid ratio. Data points represent mean ± SEM (*n* = 3, where *n* is the number of technical replicates)

To gain a better understanding of the copper salt effects, liposomes were prepared with 100 mM CuG, 100 mM CuSO_4_, or 300 mM CuSO_4_ and the level of quercetin association was determined as a function of time. The results, summarized in Fig. 3, indicate that the maximum quercetin-to-lipid ratios achieved were 0.18 mol/mol and 0.17 mol/mol when using liposomes prepared in 100 mM CuG and 300 mM CuSO_4_, respectively. The quercetin-to-lipid ratio achieved with liposomes prepared in 100 mM CuG was almost doubled that achieved with liposomes prepared in 100 mM CuSO_4_ (0.18 versus 0.10 mol/mol, respectively) (Fig. 3a). As shown in Fig. 3b, liposomes prepared in 300 mM CuSO_4_ had almost 3 times the amount of associated copper when compared to liposomes prepared in 100 mM copper solutions, regardless of the salt form. Furthermore, the amount of liposome-associated copper was essentially the same for liposomes prepared in CuSO_4_ or CuG. Finally, in all formulations studied there was no loss of associated copper following the addition of the liposomes to powdered quercetin.

**Fig. 4:**
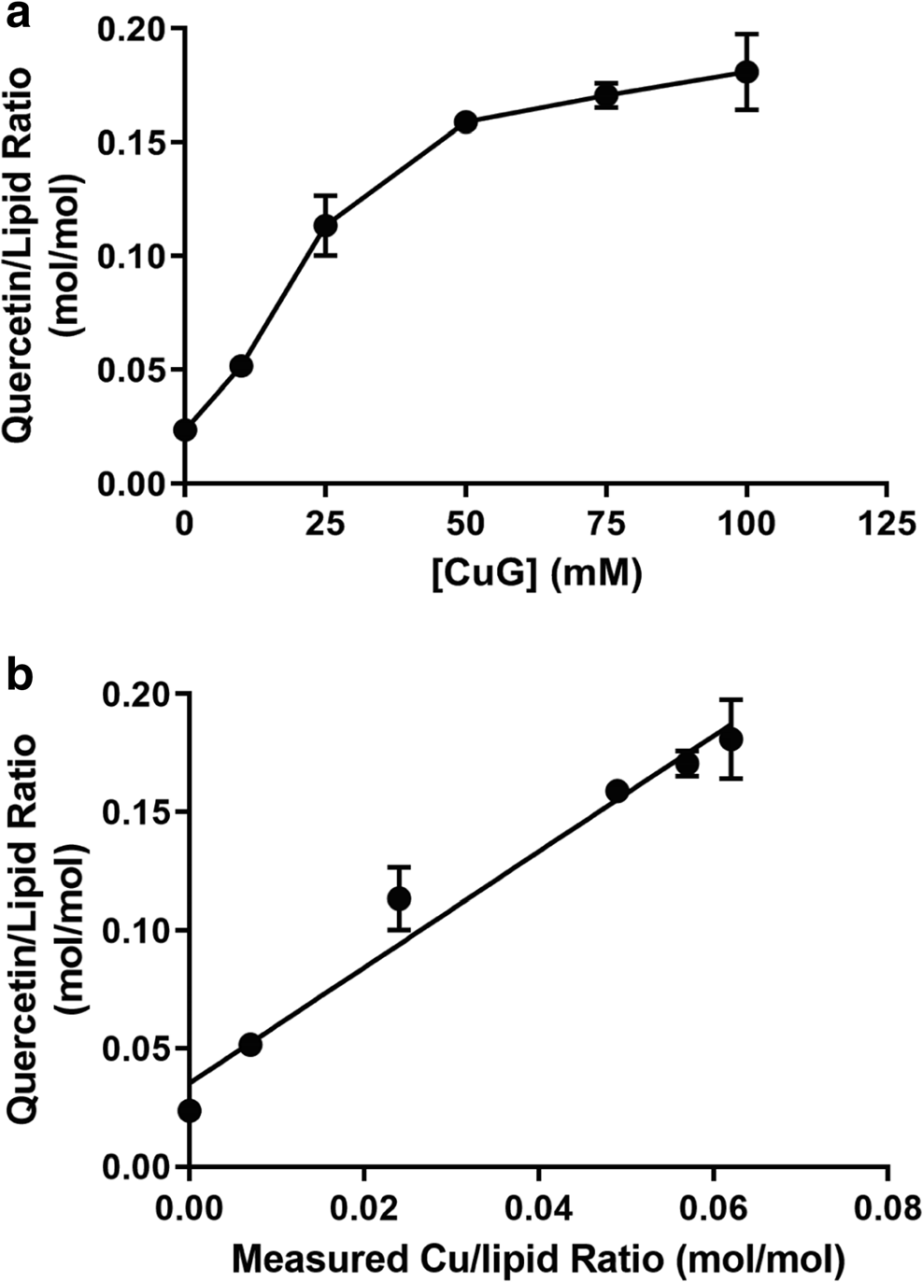
Quercetin association with liposomes prepared using solutions containing different copper gluconate (CuG) concentrations. **a** Liposomes prepared in solutions of 0, 10, 25, 75, and 100 mM CuG were mixed with powdered quercetin at 60 °C for 60 min. At that time, liposomes were passed through Sephadex G-50 spin columns (see “Materials and Methods”) and the concentration of quercetin and liposomal lipid were measured (see “Materials and Methods”) and plotted as the quercetin to lipid ratio. **b** The level of liposome-associated quercetin was plotted against the measured copper-to-lipid ratio. Copper was measured by AAS following separation of the quercetin liposomes on a Sephadex G-50 column. Data points represent mean ± SEM (*n* = 3, where *n* is the number of technical replicates)

Similar to the results obtained with copper sulfate-containing liposomes, there was a linear correlation between the quercetin association with liposomes and the amount of CuG used when preparing the liposomes (Fig. 4). The slope of the calculated quercetin-to-lipid ratio plot versus the calculated copper-to-lipid ratio (see Fig. 4b) was 2.45, suggesting that the quercetin-to-copper ratio was likely 2:1 when using liposomes prepared with CuG. In the absence of copper, the level of quercetin association with the liposomes was comparable to that measured with the CuSO_4_ control liposomes (Fig. 2a).

**Fig. 5:**
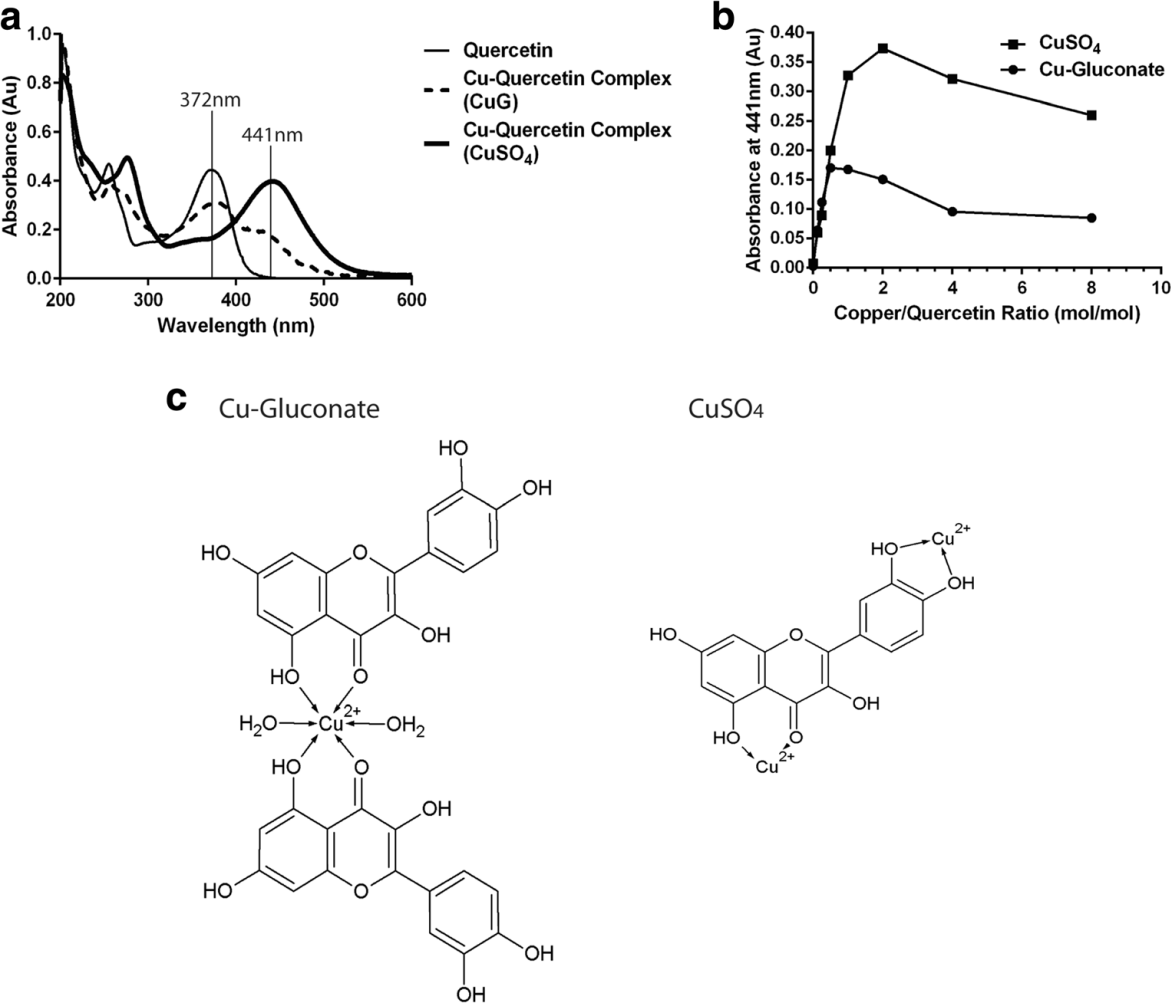
Spectrophotometric studies evaluating quercetin-copper interactions. **a** Quercetin (at a fixed quercetin concentration of 5 μg/mL in methanol) and the complexes it forms with copper can be studied spectrophotometrically, where quercetin exhibits an absorption maximum at 372 nm and the quercetin-copper complex has an absorption maximum at 441 nm, indicative of formation of a yellow complex. The shift in the absorption maximum from 372 to 441 nm was most dramatic when using copper sulfate. If copper gluconate was used, there was a reduction in absorbance at 372 nm and an increase in absorbance at 441 nm, suggestive of a more complicated interaction. **b** The absorbance 441 nm was measured in methanol solutions of quercetin (5 μg/mL) at copper-to-quercetin ratios of 1:8, 1:4, 1:2, 1:1, 2:1, 4:1, and 8:1, where copper was added as copper sulfate (filled squares) or copper gluconate (filled circles). **c** Proposed molecular structures of copper-quercetin complex when the complex is formed using copper gluconate (left panel) or CuSO_4_ (right panel)

The studies summarized above suggest that the copper-to-quercetin ratio within the formulations differed when using CuSO_4_ and CuG. The nature of this interaction was further characterized by UV absorption spectrophotometry. As shown in Fig. 5, quercetin alone exhibits an absorption UV peak at 372 nm while complexation with copper shifts the maximal absorbance to 441 nm. Interestingly, complexation with CuSO_4_ resulted in a more distinct peak at 441 nm (Fig. 5a). The increase in absorbance at 441 nm was measured when a fixed quercetin concentration was incubated with varying concentrations of copper (see “Materials and Methods”). The maximum increase in absorbance at 441 nm was achieved at copper-to-quercetin ratio of 2 (mol/mol) when CuSO_4_ was used and 0.5 (mol/mol) when CuG was used (Fig. 5b). These findings support the idea that quercetin forms a 2:1 (quercetin:copper) complex in the presence of CuG and a 1:2 (quercetin:copper) complex in the presence of CuSO_4_ (Fig. 5c).

**Fig. 6:**
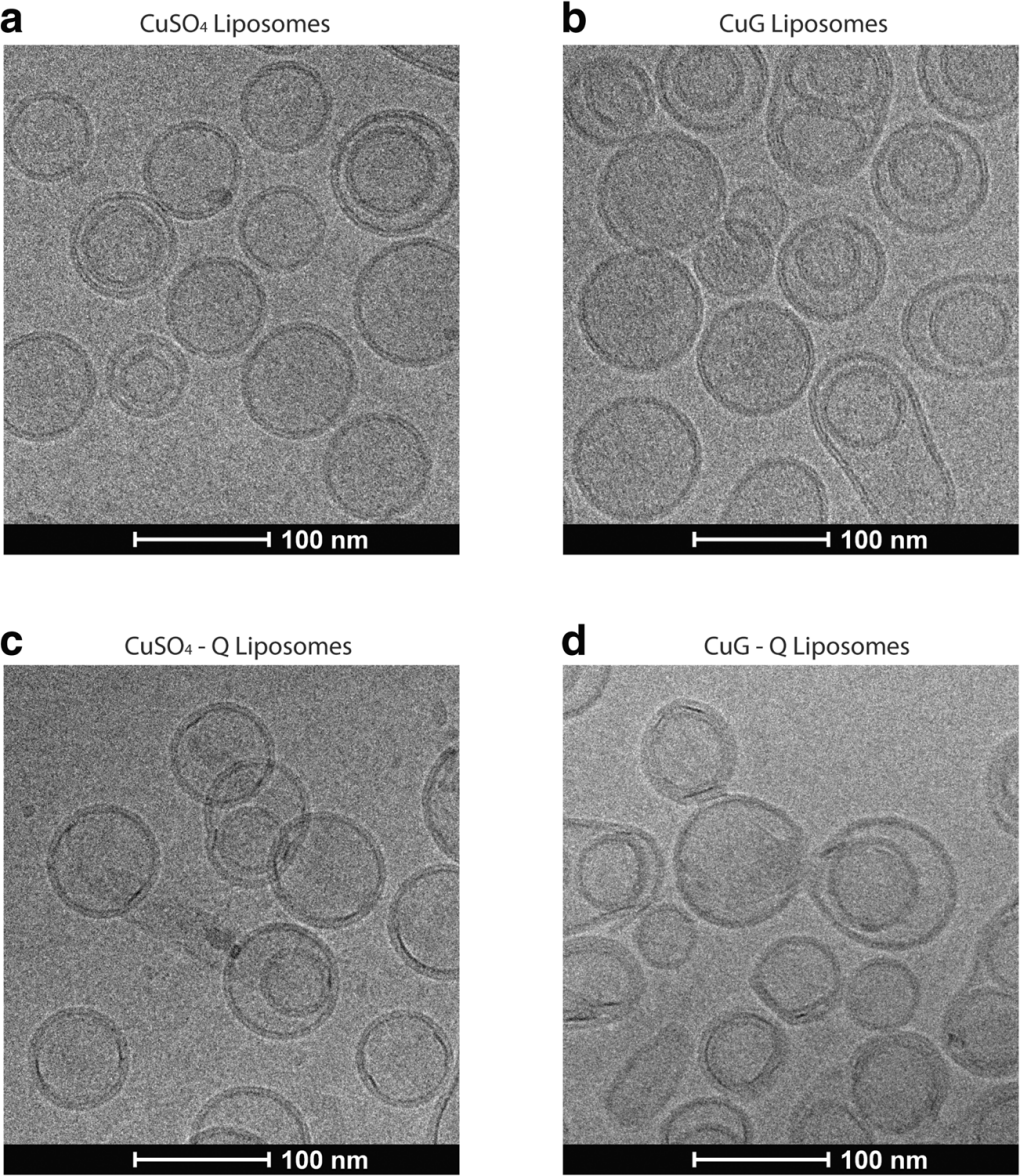
Representative cryo-transmission electron microscopy images of liposomes. The images correspond to copper sulfate (CuSO_4_)-containing liposomes (**a**), copper gluconate (CuG)-containing liposomes (**b**), CuSO_4_-containing liposomes with quercetin (CuSO_4_-Q) (**c**), and CuG-containing liposomes with quercetin (CuG-Q) (**d**). The quercetin-containing liposomes were prepared at 60 °C, incubated for 60 min. Please refer to “Cryogenic electron microscopy of copper-quercetin formulations” for the preparation of cryo-TEM images

To gain a better understanding of the morphology of the liposome quercetin formulations, liposomes with encapsulated CuG, quercetin associated with CuG-containing liposomes, liposomes with encapsulated CuSO_4_, and quercetin associated with CuSO_4_-containing liposomes were imaged by cryo-electron microscopy (CEM) (Fig. 6). The results illustrated two points: (i) the liposomes with associated quercetin in liposomes prepared using CuG or CuSO_4_ did not exhibit an electron-dense core, suggesting that if precipitates were formed, they were not electron dense; and (ii) the association of quercetin in the CuG-containing liposomes exhibited greater particle size—consistent with the particle size analysis obtained with this formulation (Table 1).

### Suitability of the copper-quercetin liposomes as a formulation for intravenous use

**Fig. 7:**
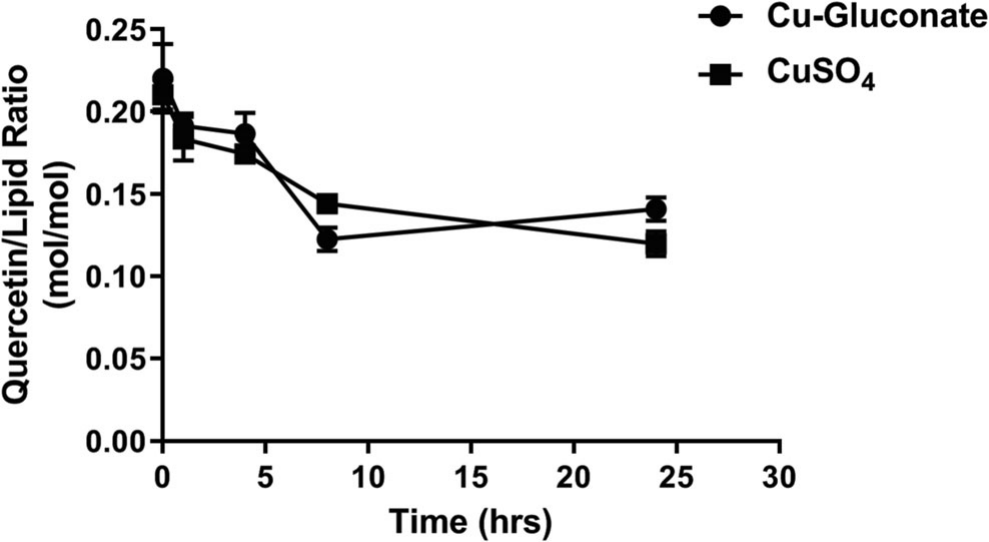
In vitro stability of copper-quercetin formulations prepared using liposomes prepared in 300 mM CuSO_4_ or 100 mM copper gluconate. The formulations were incubated in 80% FBS at 37 °C over 24 h with time points taken at 0, 1, 4, 8, and 24 h (see “Materials and Methods”). At the indicated time points, the liposomes were passed through Sephadex G-50 columns and the liposomes (in the excluded fraction) were assessed for liposomal lipid and quercetin as described in the “Materials and Methods” section. Data points represent mean ± SEM (*n* = 3, where *n* is the number of technical replicates)

The stability of the copper-quercetin formulation (one prepared with CuSO_4_ (300 mM)-containing liposomes and one prepared with CuG (100 mM)-containing liposomes) was assessed in the presence of fetal bovine serum (FBS) when incubated at 37 °C over 24 h (see “Materials and Methods”). Stability was determined by measuring time-dependent changes in the quercetin-to-lipid ratio. The results, shown in Fig. 7, indicate that there was a decrease in the quercetin-to-lipid ratio over the first 8 h under these conditions. A 17%, 25%, and 44% decrease was noted at 1, 4, and 8 h, respectively. There was not a significant change in quercetin-to-lipid ratio after 8 h and the two formulations appeared to behave similarly.

**Fig. 8:**
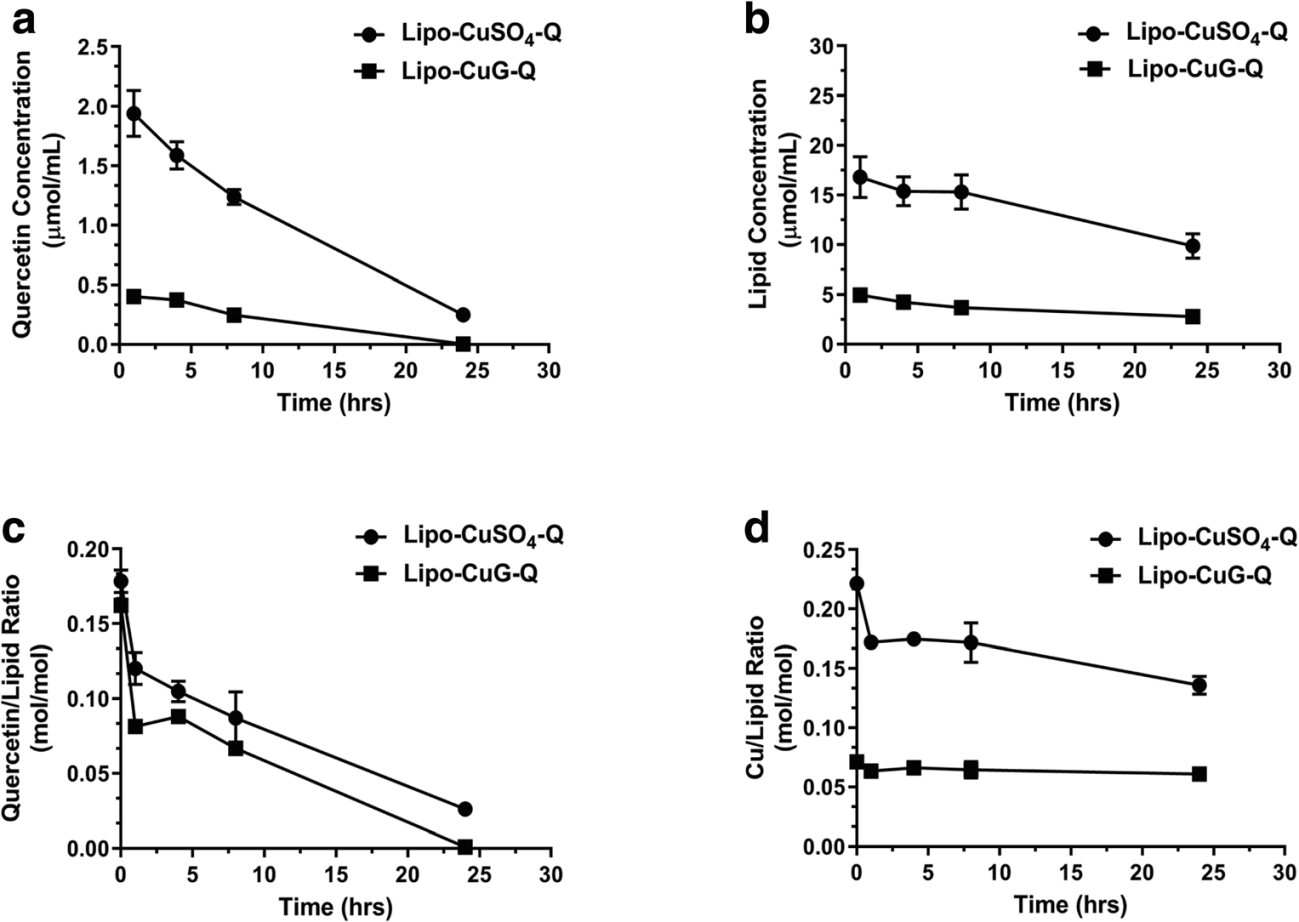
Pharmacokinetics of copper-quercetin formulations made with liposomes prepared in 300 mM CuSO_4_ or 100 mM CuG. Female RAG2m mice were injected intravenously with a single bolus dose of the indicated liposomal quercetin formulation at a quercetin dose of 50 mg/kg. Plasma concentrations of quercetin (**a**) and lipids (**b**) were measured at the indicated time points following administration. **c** Measured quercetin and liposomal lipid concentrations were used to calculate the quercetin to lipid ratio as a function of time; an assessment that estimates how fast quercetin is released from the liposomes in the plasma compartment. **d** Plasma copper levels were measured by AAS (see “Materials and Methods”) and these values were used to calculate the copper to liposomal lipid concentration in the plasma compartment; an assessment that estimates whether copper is released from liposomes in the plasma compartment. Data points represent mean ± SEM (*n* ≥ 4, where *n* is the number of mice per treatment group)

Pharmacokinetic studies were completed following the injection of the formulations into RAG2m mice (injected iv using a dose of 50 mg/kg) with the liposomal copper-quercetin formulations prepared using liposomes made with CuSO_4_ or CuG. At 1, 4, 8, and 24 h, plasma was collected and then analyzed for liposomal lipid, quercetin, and copper as described in the “Materials and Methods.” The results, summarized in Fig. 7, indicate that the formulation prepared with CuG-containing liposomes was eliminated more rapidly than formulations prepared using CuSO_4_ and this was consistent with the increase in particle size and polydispersity observed when using quercetin formulations prepared using CuG—containing liposomes. Analyses of the pharmacokinetics data with PK Solutions software (Summit Research Services, USA) revealed that the area under the curve (AUC) is 8382 μg h/mL for the quercetin formulations prepared using liposomes made with copper sulfate and 1577 μg h/mL for quercetin formulations prepared using liposomes made with CuG (Table 2). These results highlight two important points: (i) the rate of quercetin release from the liposomes in the plasma compartment is comparable for the two different formulations (Fig. 8c), albeit a more substantial initial loss of associated quercetin was observed when using the formulation prepared with copper gluconate-containing liposomes and (ii) the results suggest that encapsulated copper is retained within the circulating liposomes for both formulations (Fig. 8d), suggesting that quercetin is released from the liposomes rather than a copper-quercetin complex.

**Table 2:** Pharmacokinetics parameters of flavopiridol following intravenous administration (5 mg/kg)

	*T*_1/2_ (h)	AUC (μg h/mL)	*V*_d_ (mL)	CL (mL/h)
Lipo-CuSO_4_-Q	6.91	8382	1.76	0.18
Lipo-CuG-Q	2.47	1577	3.73	0.91

### Therapeutic potential of quercetin when used in combination with irinotecan

**Fig. 9:**
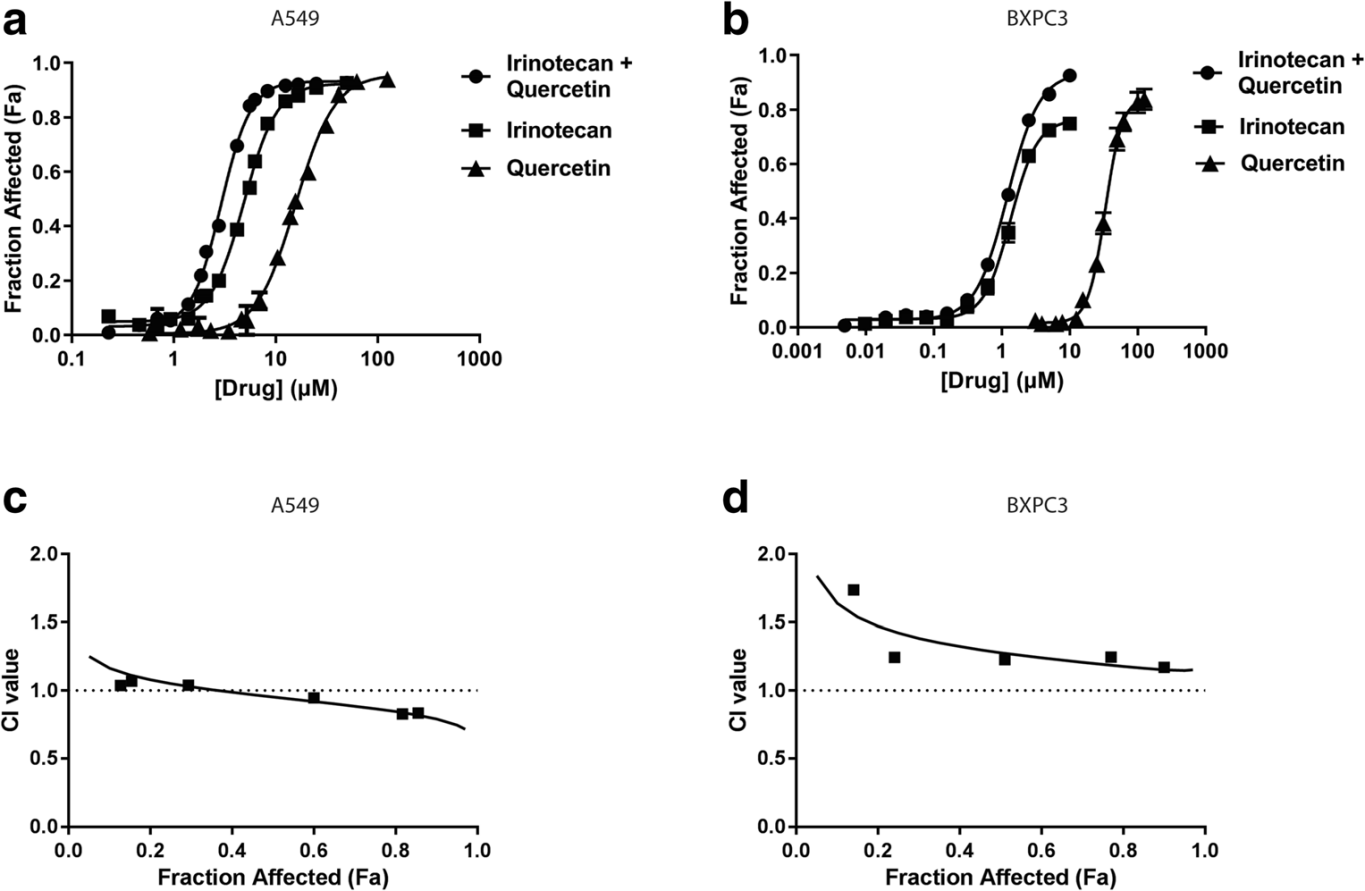
In vitro *assay* assessing the cytotoxic effects of quercetin alone and in combination with irinotecan. The quercetin (Quer) and/or irinotecan (CPT11) dose-response curves determined for A549 lung cancer cells (left panel) and BxPC3 pancreatic cancer cells (right panel) using a imaging method reliant on the IN Cell Analyzer as described in the “Materials and Methods” section. For combination effects, quercetin and CPT11 were added at ratios of 1:2.5 (CPT11:Quer) for A549 and 1:18 (CPT11:Quer) for BxPC3. The dose-response curve for the combination was plotted based on CPT11 concentrations (**a**). Combination indices (CI) derived from the dose response of the combination treatments are plotted against the fraction of affected (F_a_) cells, where a F_a_ of 1 indicated 100% cell kill. CI > 2: antagonistic 2 > CI > 0.8: additive, and CI < 0.8: synergistic. **b** Data points represent mean ± SEM (*n* ≥ 3, where *n* is the number of independent experiments)

Based on previous studies, it was concluded that quercetin combined with irinotecan (CPT-11) could produce synergistic cytotoxic effects [26–28]. Our group has developed a liposomal CPT-11 formulation (Irinophore C) with significant therapeutic activity [29, 30] and this gives us a favorable position to develop a combination product comprising CPT-11 and quercetin. For this reason, we choose to assess the combination effects achieved when using these drugs in combination. Consistent with previous reports, quercetin-induced cytotoxic effects were only achieved when using micromolar concentrations of the agent: the IC_50_ of quercetin was 22 and 37 μM when added to A549 and BxPC3 cells, respectively (Fig. 9a, b). When used in combination with CPT-11, quercetin and CPT11 displayed minimal synergy at high effect levels (> 60% cell kill) for A549 but the two agents acted additively or antagonistically at all effect levels when used to treat BxPC3 cells (Fig. 9c, d). These data did not provide sufficient justification to further pursue quercetin as a single agent or in combinations in vivo.

## Discussion

Others have highlighted that quercetin has anticancer activity and it has been argued that it can act as an apoptosis promoter through modulations of proteins such as Bcl-2 and Akt-1 [3, 7, 8]. Unfortunately, since the water solubility of quercetin is poor, its oral bioavailability is limited [10, 31] and its therapeutic utility is limited because of this. For example, a single 4-g oral dose of quercetin resulted in no detectable plasma or urine concentrations of quercetin and less than 1% of the quercetin was absorbed unaltered in the gastrointestinal tract [32]. Importantly, this is not the only concern when considering the development of quercetin as a therapeutic to treat cancer. Most studies have shown that quercetin can only induce anticancer effects when present in high micromolar (10–20 μM) concentrations [33, 34]. If a suitable intravenous formulation of quercetin was developed, issues related to bioavailability would then be addressed and perhaps suitable concentrations of quercetin in the blood could be obtained. We recently described a formulation method for poorly soluble drugs, including flavonoids like quercetin, that considers the use of compounds that can coordinate metals and nanotechnology [23]. In this report, liposomal formulations prepared based on quercetin-copper binding have been characterized. The results suggest that the encapsulated copper salt can influence the copper-quercetin complex that is formed. Importantly, the formulations made exhibit more than 100-fold improvements in apparent solubility and are suitable for intravenous administration.

Quercetin is difficult to prepare as an iv-injectable formulation. When described, these formulations rely on organic solvents such as dimethyl sulfoxide (DMSO) which, when used, have been shown to be toxic at many levels [35]. Therefore, it is not surprising that efforts have been invested to develop alternative quercetin formulations. For example, when compared to free quercetin, liposomal quercetin formulations often displayed improved blood circulation and decreased rates of elimination from the plasma compartment [24, 25, 36]. In these previous studies, quercetin was formulated into liposomes passively when the liposomes are first made. Passive encapsulation into liposomes is very inefficient and requires preparation of liposomes at high lipid concentrations [37]. In contrast, the novel copper-based methodology described here was extremely efficient, where encapsulation amount was dependent on the copper-to-quercetin ratio formed. The copper-based technology resulted in more stable and consistent formulations of quercetin. The CuSO_4_-based formulation showed improved pharmacokinetics compared to liposomal formulations previously described in literature with a 3-fold increase in circulation half-life (2 h versus 0.5 h) and an 18 fold increase in total drug exposure (AUC) (1.94 versus 0.106 μmol/mL) [24]. This copper-based formulation was well-tolerated at the current maximum feasible injected dose in RAG2m mice with no acute or chronic (14 days) toxicities observed (data not shown). Additionally, necropsy reports of the animals used to assess toxicity studies were unremarkable.

In addition to liposomes, biofunctional metal-phenolic films have also been previously investigated to overcome the solubility challenges of quercetin [38]. Bertleff-Zieschang et al. described one-step, pH-dependent coordination of quercetin with Fe^III^ ions to form thin films and hollow capsules on solid surfaces [38]. The formation of these capsules and films helped address the poor aqueous solubility of free flavonoids like quercetin [38]. In this regard, the hollow iron-quercetin capsules are similar to our liposomes as they also provide a solution to solubilize quercetin. However, our liposomes present a few advantages, most notable of which is the physical shielding effects that the phospholipids of the liposomes are able to provide for the encapsulated content. As nanocarriers, liposomes are well known to act as steric barriers that may discourage interactions between entrapped compounds and plasma proteins [39]. This property is particularly attractive for quercetin as this flavonoid is known to extensively bind human plasma protein and it has been suggested that this interaction results in poor cellular availability of quercetin [40]. By encapsulating quercetin in liposomes, we would able to curb this activity-hindering interaction with plasma proteins. Additionally, although Bertleff-Zieschang et al. did not evaluate the pharmacokinetic profile of the hollow capsules, we believe that by shielding quercetin within liposomes, our formulation would also have improved pharmacokinetics, namely circulation time and total drug exposure, when compared to the hollow capsules of quercetin. This improved pharmacokinetic profile could enhance quercetin’s clinical value as quercetin is known to be a substrate of P-glycoprotein and its absorption rate is limited by high efflux rate [41].

Metal-based therapeutics made a major leap in the 1970s with the emergence of platinum-based complexes such as cisplatin, carboplatin, and oxaliplatin as widely successful, and potent therapeutics [42]. However, efforts have been made to identify alternatives to replace platinum-based therapeutics primarily due to the many crippling toxicities that these drugs engender and copper-based therapeutics emerged as a potential replacement candidate [43]. One of the primary reasons that copper was selected as the metal of choice was its role as an essential metal. Unlike platinum, which is not native to the human body, copper is an important trace element in the growth and development of all aerobic organisms and plays a crucial role in a number of biological pathways [42, 44]. As such, copper is tightly regulated and its homeostasis is rigorously maintained to avoid copper-related toxicities [44, 45]. In the blood, plasma protein carriers such as albumin and ceruloplasmin transport the copper absorbed into the bloodstream to the liver and the kidney for excretion and distribution [46]. On the cellular level, copper transporters such as CTR1 and copper efflux pumps like ATP7A/B aid in the regulation of cytosolic copper levels [42]. The presence of these intricate regulatory mechanisms helps attenuate toxicities that copper-based therapeutics may have otherwise engendered, allowing copper-based drugs to be better tolerated. In addition to having natural regulatory biological pathways, copper was also chosen as the metal of preference due to its potential to enhance anticancer activities. Disulfiram, an FDA-approved drug for treating alcoholism, paints a perfect picture of a copper-activated agent. As an anticancer agent, disulfiram has shown to have minimal anticancer activity in the absence of copper but is active in the nanomolar range against various cancer cell lines in the presence of copper [47, 48]. In the case of quercetin, forming a complex with copper did not enhance the activity of quercetin (data not shown). However, the use of copper still solved the low solubility problem that has long prevented the use of flavonoids such as quercetin in a clinical setting. Here, we have provided proof-of-concept data using copper, but we do not preclude the possibility of using other metals, such as zinc. In agreement with numerous published studies [17, 49, 50], our data suggested that quercetin forms a 2:1 ligand-to-metal complex with copper (II) ions in the presence of CuSO_4_ and a 1:2 ligand-to-metal complex with copper (II) ions in the presence of CuG (Fig. 5b). The different complexes formed in the presence of CuSO_4_ versus CuG help explain the different encapsulation efficiencies observed. Gluconic acid forms strong complexes with many transition metals including copper [51]. The presence of a competitive copper chelator would result in the formation of a different copper complex than what is seen with CuSO_4_. Thus, in the presence of CuG, two quercetin molecules were needed to bind with copper ions to engender dissociation of copper ions away from gluconic acid. In contrast, sulfate does not form a complex with copper ions and the quercetin molecule can bind to two copper (II) ions. Therefore, when considering equivalent Cu (II) concentrations, CuG-containing liposomes appear to facilitate improved quercetin association compared to CuSO_4_-containing liposomes. However, CuG-quercetin liposomes were cleared from the plasma compartment much more rapidly compared to CuSO_4_-quercetin liposomes. This may have been the result of the greater variation in particle size as shown in the CEM images of CuG liposomes (Fig. 6) and the increase in particle size seen when using CuG—containing liposomes with encapsulated quercetin. The rate of liposome elimination is increased as the size of the liposome increases [52].

Regardless of the binding interactions, quercetin and copper-quercetin complexes are poorly soluble in aqueous solutions and this is one factor that limits quercetin’s therapeutic potential. Many studies have demonstrated that as a single agent, quercetin is not a potent chemotherapeutic agent, with effective doses at micromolar concentrations [5, 53–55]. Therefore, it can be argued that quercetin’s therapeutic value may lie in combination therapy. In fact, it has previously been shown that when used in combination, quercetin potentiates existing chemotherapeutic agents such as cytosine arabinoside, doxorubicin, and irinotecan for treatment against leukemia, breast cancer, and gastrointestinal cancer, respectively [26, 56, 57]. In particular, quercetin has been shown to work well in combination with irinotecan, demonstrating promise in the treatment of advanced non-small-cell lung cancer, with an overall response rate of up to 32% [58]. In an initial in vitro screen, when quercetin was added concurrently with irinotecan, the interaction between the two drugs suggested additively (Fig. 9). The difference in the tissue origin and the genetic profiles between the cell lines may have accounted for this. Regardless, further studies to elucidate the therapeutic utility of quercetin combinations and to identify suitable candidates to use in combination with quercetin are needed. More studies are needed to define how a liposomal copper-quercetin formulation would be used in combination in preclinical animal models. Additionally, the use of copper complexation methods to encapsulate quercetin acts as a proof-of-concept and will now allow us to further explore utility of this copper-based technology with other promising flavonoids or flavonoid-like candidates that share similar copper-chelating properties as quercetin.

## Conclusions

In summary, we have successfully addressed the solubility issues associated with quercetin by generation of a novel liposomal formulation of copper-quercetin. Compared to other quercetin-based formulations previously described, our formulation takes advantage of the well-characterized propensity of liposomes to enhance the circulation longevity of an associated drug. Continued efforts will be made to explore and evaluate quercetin’s value as a component in combinations for the treatment of cancer.
